# Long term outcomes following embolisation of bronchial and non-bronchial systemic arteries for the management of haemoptysis – a 20-year experience

**DOI:** 10.1186/s42155-025-00551-0

**Published:** 2025-06-18

**Authors:** Shyamal Patel, Lucy Rose Howroyd, Helen Bucknall, Hussain Memon, Robert Morgan, Joo-Young Chun

**Affiliations:** George’s University Hospitals NHS Foundation Trust, Blackshaw Road, London, SW17 0QT UK

**Keywords:** Bronchial artery embolization, Haemoptysis, Aspergilloma

## Abstract

**Background:**

Bronchial artery embolisation (BAE) is considered the most effective non-surgical technique for management of moderate-massive haemoptysis. Associated risks include neurological compromise such as stroke and spinal cord ischaemia. We aim to evaluate post-procedural outcomes and complication rates.

**Materials and methods:**

A single-centre retrospective observational study was conducted for BAE cases performed between January 2002-June 2022 in a London teaching hospital. Data was collected from electronic medical records and Picture Archiving Communications System (PACS). Primary outcomes were measured, and statistical analysis was performed to identify risk factors for haemoptysis recurrence.

**Results:**

One hundred eleven patients underwent 141 procedures with technical success achieved in 87.8% and clinical success in 84.8%. The most common causes of haemoptysis were aspergilloma (24.8%), bronchiectasis (19.1%) and malignancy (11.3%). Haemoptysis recurrence occurred in 65 cases (46%) with 20 patients undergoing repeat embolisation. Aspergillosis, cystic fibrosis, and non-tuberculous pneumonia were identified as risk factors for recurrent haemoptysis (*p* < 0.005). Pre-procedure MDCTA did not improve technical success. The rate of stroke in the cohort was 6.4% (9 cases), which is more so than quoted in the literature. Four of these patients presented with apical cavitations secondary to infection (aspergilloma or bacterial pneumonia).

**Conclusions:**

BAE is an effective endovascular treatment in patients with massive and recurrent haemoptysis. However, there is a well-documented risk of recurrent symptoms and early mortality, particularly in the setting of aspergilloma, cystic fibrosis and non-tuberculous pneumonia. The risk of stroke should not be underestimated. Patients should be counselled appropriately during informed consent prior to embarking on BAE.

## Introduction

Massive haemoptysis is a respiratory emergency associated with significant mortality estimated to be above 50% [1, 2]. Bronchial artery embolisation (BAE) is a minimally-invasive endovascular technique considered to be the most effective non-surgical procedure for control of moderate to massive haemoptysis [3–5].

Technical success rates following BAE are estimated between 70–99%. However, recurrence of haemoptysis is well documented in up to 57.5% and definitive management of the underlying cause is essential to prevent further episodes [3, 4]. The most common complication associated with BAE is chest pain, reported in up to 34.5% of cases. It is mostly transient and requires no further intervention. Neurological complications include stroke and spinal cord ischaemia. They are less common with reported rates of 2% and 4.4% respectively and are thought to be due to non-target embolisation [4].

Haemoptysis can occur due to a variety of pulmonary, vascular, and haematological causes and the culprit bleeding vessels may arise from the bronchial arterial tree or other systemic arteries, most commonly intercostal, subclavian, inferior mammary and inferior phrenic arteries [4]. The Cardiovascular and Interventional Radiological Society of Europe (CIRSE) standards of practice document for BAE (2022) highlights the practical benefits of performing MDCTA prior to BAE [6]. This includes identifying an underlying cause of haemoptysis, confirming the presence of enlarged bronchial and non-bronchial systemic arteries, as well as reviewing arterial access [4, 6–15].

Despite these potential advantages, pre-procedural MDCTA is not universal practice. MDCTA has been reported as having up to 97% concordance with transcatheter angiography in identifying bleeding vessels [11]. However, the impact of pre-procedural MDCTA on technical and clinical outcomes of BAE remains equivocal [4, 12, 16]. One case series demonstrated pre-procedural MDCTA increased the technical success rate of BAE [17], while another study suggested MDCTA bears no influence on the clinical success of BAE [16]. The latter study argued that MDCTA is not a necessity and should not delay prompt therapeutic embolisation.

The aim of this study is to evaluate the immediate and long-term outcomes of BAE with particular interest in identifying demographic or clinical risk factors that predict poorer outcomes. Neurological complications following BAE may have devastating consequences and thus this study aims to identify their incidence as well as any predictive factors or mechanisms which would increase their likelihood. Finally, this study aims to evaluate the role of pre-procedural MDCTA and its impact on technical or clinical outcomes of BAE.

## Materials and methods

A retrospective review was conducted in all patients who underwent BAE over a 20-year period (January 2002 and June 2022) at a tertiary referral centre in London, United Kingdom. BAE was performed in adult patients with moderate to massive haemoptysis or recurrent haemoptysis that was refractory to medical management. Ethical approval was sought but waived for this retrospective study. This study cohort includes 50 patients whose outcomes were previously published [5].

Patient medical records and relevant imaging were reviewed to extract data on demographics, aetiology of haemoptysis, concurrent medical treatments, arteries embolised during BAE and corresponding clinical outcomes. Primary outcomes were technical success (defined as successful embolisation of target arteries to stasis), clinical success (defined as cessation of haemoptysis during the same hospital admission), peri-procedural complications, and time to haemoptysis recurrence.

Secondary outcomes included identification of risk factors for clinical failure or early symptom recurrence. This was defined as recurrent moderate or massive haemoptysis as reported by patients and/or requiring admission to hospital. In addition, the impact of pre-procedural imaging on technical and clinical outcomes was evaluated. The phase of CT contrast enhancement and visibility of target bronchial and/or non-bronchial arteries were assessed, and these were compared with corresponding catheter angiographic images. Cumulative non-recurrence rates were analysed using the Kaplan–Meier method. Statistical analysis using a Fisher’s exact test was performed to identify any demographic or clinical factors related to recurrent symptoms.

### Procedural technique

**Fig. 1:**
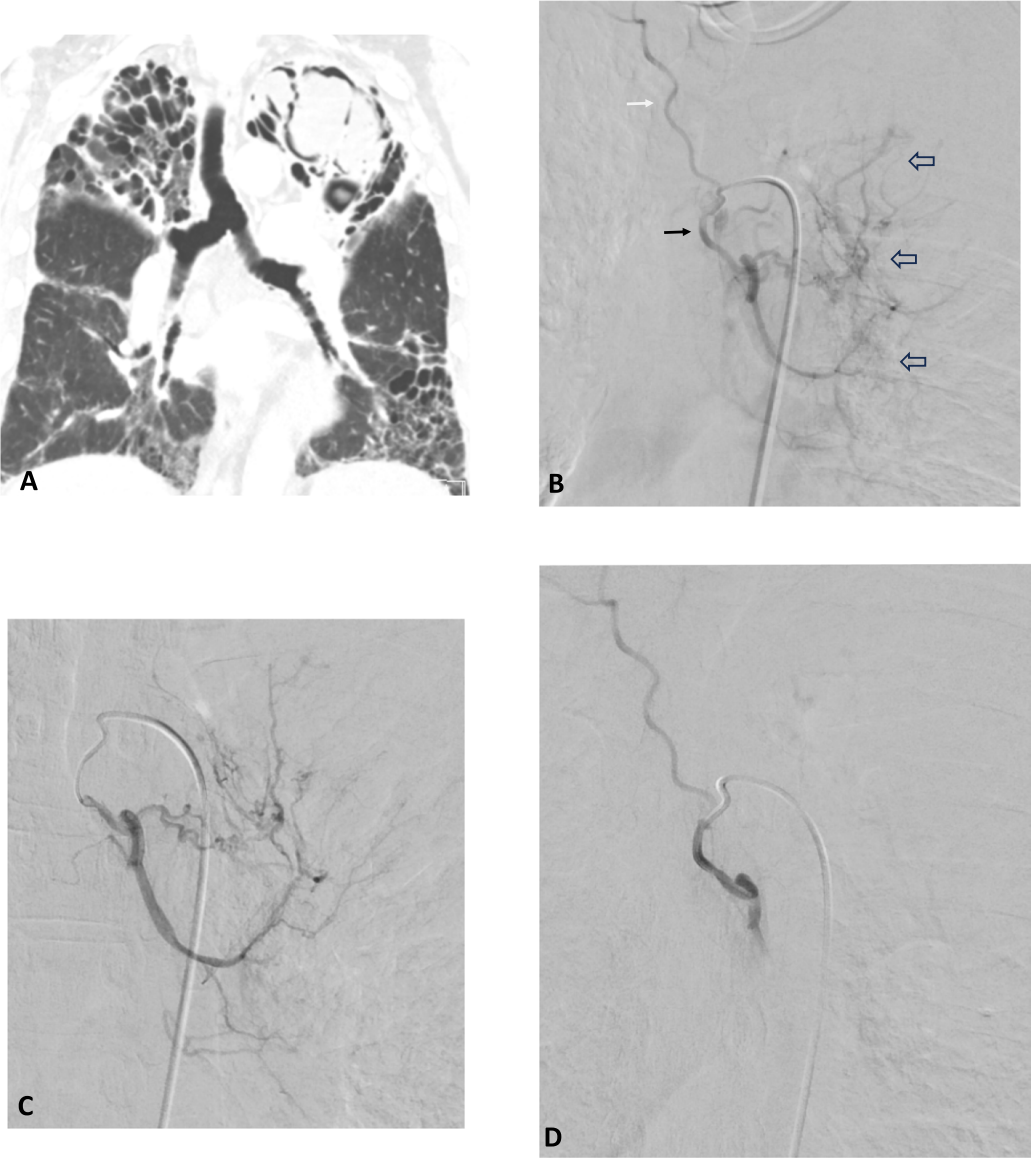
63-year-old man with stage IV sarcoidosis presenting with recurrent haemoptysis. **A** Coronal CT image shows bilateral upper lobe fibrosis and a large left apical aspergilloma. **B** Selective left bronchial angiogram of common trunk of R intercostal (white arrow) and L bronchial artery (black arrow). A leash of abnormal branches (open arrows) arising from an enlarged L bronchial artery. **C** Super-selective L bronchial angiogram prior to embolisation with ns-PVA. D) Completion angiograms shows distal pruning of L bronchial branches and preserved opacification R intercostal artery

The technique of BAE has been previously described in detail [5]. All procedures were performed or supervised by an experienced consultant Interventional Radiologist. Arterial access was obtained in the right common femoral artery and a flush thoracic aortogram was performed to identify the origin and anatomy of bronchial and non-bronchial arteries. Selective catheterisation was performed with an appropriately shaped 4 - 5 Fr catheter. If the bronchial arteries were normal, non-bronchial systemic vessels (intercostal, subclavian, internal mammary, inferior phrenic, arteries) were evaluated. If a more distal position was required to facilitate safe embolisation (i.e. avoid important side branches or provide a more stable position for embolisation), the target vessels were super-selected with a 2.4/2.7 Fr microcatheter (Fig. 1). If technically possible, all abnormal vessels were embolised to stasis. The preferred embolic agent was 355–500 micron non-spherical polyvinyl alcohol (ns-PVA) particles (Contour—Boston Scientific, Marlborough, Massachusetts).

## Results

### Patient demographics

The cohort consisted of 158 procedures performed in 127 patients. 16 patients were lost to follow up and excluded from the analysis leaving 141 procedures in 111 patients. Sixty-four patients were males and 47 were female, with a mean age of 55.6 years (range 19–87, standard deviation 16.5). Twenty patients underwent more than one BAE procedure, of whom 15 had 2 procedures, 3 patients had 3, and 2 patients had more than 3 procedures. The underlying causes of haemoptysis are outlined in Table 1 with the most common being aspergilloma (24.8%), bronchiectasis (19.1%), pulmonary malignancy (11.3%) and non-tuberculous pneumonia (9.2%).

**Table 1:** Causes of haemoptysis

Aetiology	Number of patients (%)	Number of procedures (%)
Bronchiectasis	23 (17.4)	27 (15.3)
Aspergilloma:	21 (15.9)	35 (19.9)
* Secondary to* *Sarcoidosis*	*8 (6.1)*	*16 (9.1)*
* Secondary to* *Tuberculosis*	*13 (9.8)*	*19 (10.8)*
Idiopathic	18 (13.6)	23 (13.1)
Malignancy	16 (12.1)	16 (9.1)
Active TB	9 (6.8)	9 (5.1)
Pneumonia (non-TB)	9 (6.8)	13 (7.4)
COPD	5 (3.8)	5 (2.8)
Cystic Fibrosis	3 (2.3)	5 (2.8)
Pulmonary HTN	3 (2.3)	4 (2.3)
Vasculitis	2 (1.5)	2 (1.1)
BA aneurysm	1 (0.8)	1 (0.6)
Iatrogenic	1 (0.8)	1 (0.6)
TOTAL	111 (100)	141

### Technical & clinical success

The technical success rate with embolisation of all target arteries was 87.9% which translated to a clinical success rate of 84.4%. Super-selective catheterisation and embolisation was performed in 44/141 (31.2%) of cases, which reflects cases performed after 2004 when microcatheters became readily available.

The most employed embolic agent was 355–500-micron ns-PVA in 91.5% of procedures. In two cases, ethylene vinyl alcohol (EVOH, Onyx—Medtronic, Santa Rosa, CA) was utilised. Other agents used in a single case each include 500–710-micron ns-PVA, 900-micron spherical PVA, and a combination of 355–500-micron ns-PVA and n-butyl cyanoacrylate (NBCA). As these alternative embolics were utilised so infrequently, it was not possible to draw any conclusions regarding outcome from their use. The commonest reason for technical failure was inability to engage the origin of the bronchial arteries (29.4%), which often arise at acute angles and from the inferior surface of the aortic arch.

### Haemoptysis recurrence

The mean follow-up period was 41 months (range 0 days – 244 months). The period of 0 days follow-up pertains to two patients who underwent a technically unsuccessful procedure and died subsequently from massive haemoptysis.

**Fig. 2:**
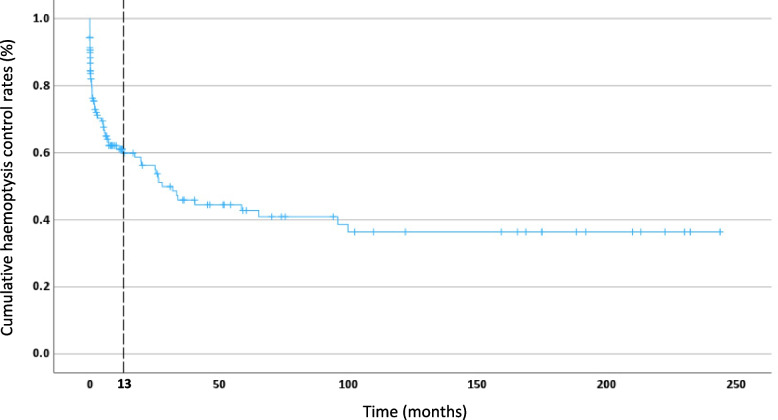
Cumulative haemoptysis control rates following BAE, calculated using the Kaplan–Meier method

Overall, there were 65 cases of recurrent haemoptysis (46%) including 6 deaths due to massive haemoptysis (4.3%). The remaining 76 patients were free of haemoptysis (54%) of whom 32 (22.7%) died during follow-up from unrelated causes. Cumulative haemoptysis control rates are depicted in Fig. 2. From the cohort, 75% of patients were free of recurrence at 1 month, 58% at 1 year, 43% at 3 years and 40% were free of recurrent haemoptysis at 5 years. The curve demonstrates majority of patients experience recurrent symptoms within the first 13 months following BAE.

Table 2 compares patients who developed recurrent haemoptysis with those who remained asymptomatic. Analysis revealed that aspergilloma, cystic fibrosis and non-tuberculous pneumonia were statistically significant risk factors for symptom recurrence (*p* < 0.05).

**Table 2:** Risk factors associated with recurrent haemoptysis

	No recurrence (%) *n* = 76	Recurrence (%) *n* = 65	*P* value
Gender:			
M	44 (57.9)	35 (53.8)	0.74
F	32 (42.1)	30 (46.2)	
Aetiology:			
Active TB	8 (10.5)	1 (1.5)	0.99
Aspergilloma	13 (17.1)	22 (33.8)	**0.018**
Bronchial artery aneurysm	1 (1.3)	0 (0)	1
Bronchiectasis	16 (21.1)	11 (17)	0.8
Cystic Fibrosis	0 (0)	5 (7.7)	**0.019**
Emphysema	4 (5.3)	1 (1.5)	0.96
Iatrogenic	1 (1.3)	0 (0)	1
Idiopathic	16 (21.1)	7 (10.8)	0.97
Malignancy	10 (13.2)	6 (9.2)	0.93
Pneumonia	3 (3.9)	10 (15.4)	**0.02**
Pulmonary Hypertension	2 (2.6)	2 (3.1)	0.63
Vasculitis	2 (2.6)	0 (0)	1

### Complications

Eighteen complications were recorded in total (12.8%), all of which were identified within the first 30 days following the procedure. Eight (5.7%) of these included transient chest pain which did not require any active management. Ten were grade 4–6 according to the CIRSE Classification of Complications [18]. These included 9 cases of ischaemic stroke (6.4%)—six of these patients presented with florid infection as the underlying cause of haemoptysis, 4 of whom had ipsilateral or bilateral apical lung cavitations. There were no cases of paraplegia or spinal cord ischaemia. A single patient (0.7%) developed a pseudoaneurysm at the femoral artery access site requiring surgical repair.

### Pre-procedural contrast-enhanced CT (CECT)

During 2009, there was a major PACS server storage issue at our institution and as a result, imaging studies were unavailable for review in 19 cases. Of the remaining 122 cases, pre-procedural contrast-enhanced CT scan was performed in 71 cases. These examinations were reviewed by the first and last authors independently. Table 3 outlines the type of contrast-enhanced CT examination, visibility of target arteries, and technical success rates when target arteries were visible compared with when they were not visible.

**Table 3:** Phase of contrast-enhanced CT scan, visibility of target arteries and impact on technical success (CECT – contrast-enhanced CT)

Phase of CECT	Number	Target artery visible (%)	Technical success when target artery visible on CT (%)	Technical success (%) when target artery not visible on CT (%)
Pulmonary Angiographic	24	14 (58.3%)	14/14 (100%)	8/10 (80%)
Arterial	35	31 (88.6%)	24/31 (77.1%)	3/4 (75%)
Portal venous	12	7 (58.3%)	6/7 (85.7%)	5/5 (100%)
Total	71	52	44/52 (85.0%)	16/19 (84.2%)

As expected, the optimal phase of contrast enhancement to visualise target bronchial and non-bronchial arteries was the arterial phase—88.6% were visible compared to 58.3% in both pulmonary arterial and portal venous phases. Visibility of target arteries on pre-procedure CT did not improve the likelihood of technical success of BAE in all three phases. Overall technical success was achieved in 108/122 (88.5%) of cases where pre-procedural imaging was available. These include 52 cases where the anatomy of the bronchial arteries was visible on pre-procedure CT but also 70 cases where it was not – either because a CT scan was not performed, or target arteries were not visible on CECT.

Conversely there were case examples where MDCTA proved useful. For example, an initial BAE was technically unsuccessful as catheter angiograms demonstrated no target for embolisation. Persistent haemoptysis prompted MDCTA which demonstrated abnormal collaterals arising from the internal mammary artery. The patient subsequently underwent a repeat attempt to target these non-bronchial systemic collaterals and were successfully embolised.

## Discussion

### Haemoptysis control & recurrence

The findings of this study, support the role of BAE as an effective management option in massive and recurrent haemoptysis. The clinical success and recurrence rates in this study of 84.4% and 46% are comparable to the literature where they range from 67 to 100% and 9.8% to 57.5%, respectively (Table 4) [5, 19–44]. High recurrence rates emphasise the need to manage and control the underlying disease process concurrently to limit ongoing recruitment of bronchial and non-bronchial vessels that may lead to future episodes of haemoptysis.

**Table 4:** Published BAE outcomes to date

First author, year of publication	Number of patients (n)	Immediate clinical success rate (%)	Recurrence rate (%)	Major complication rate (%)
Remy, 1977 [19]	104	84	28.6	0.9
Uflacker, 1985 [20]	64	76.6	21.4	0
Rabkin, 1987 [21]	306	90.8	33.7	0
Ramakantan, 1996 [22]	140	73	27.1	1.4
Goh, 2002 [23]	134	81.6	15.5	0
Swanson, 2002 [24]	54	94.4	24.1	0
Van den Heuvel, 2007 [25]	75	67	47	4.3
Chan, 2009 [26]	167	95.7	45	1.2
Chun, 2010 [5]	50	86	28	2
Shin, 2011 [27]	169	96.4	30.6	0
Anuradha, 2012 [28]	58	86.2	25.9	1.7
Hwang, 2013 [29]	72	93.1	40.3	0
Agmy, 2013 [30]	348	95	9.8	0.6
Pei, 2014 [31]	112	86.6	24.1	0
Fruchter, 2015 [32]	52	92	57.5	6.6
Bhalla, 2015 [33]	334	93.5	12.6	0
Shao, 2015 [34]	344	96	39.2	0.9
Tom, 2015 [35]	69	82	30	1
Dabo, 2016 [36]	67	98.5	37.3	0
Pathak, 2016 [37]	50	100	48	4
Ayx, 2016 [38]	34	94	15	3
Springer, 2018 [39]	30	93	57	0
Lee, 2019 [40]	33	100	24	0
Shimora, 2019 [41]	52	100	33	0
Martin, 2020 [42]	242	82	25	5.2
Dorji, 2021 [43]	184	70.1	48.9	6
Lu, 2022 [44]	69	92.8	34.8	0
Our study, 2025	**111**	**84.4**	**46.1**	**7.1**

Risk factors for recurrent haemoptysis have been previously suggested and include incomplete embolisation, vessel spasm and aspergilloma as the underlying cause [7]. In this cohort there was a statistically significant risk of recurrence associated with aspergilloma, cystic fibrosis and non-tuberculous pneumonia. Aspergilloma was the most common aetiology in the cohort (21 patients) accounting for 24.5% of cases and may in part reflect the significant rate of recurrence [7, 25].

**Fig. 3:**
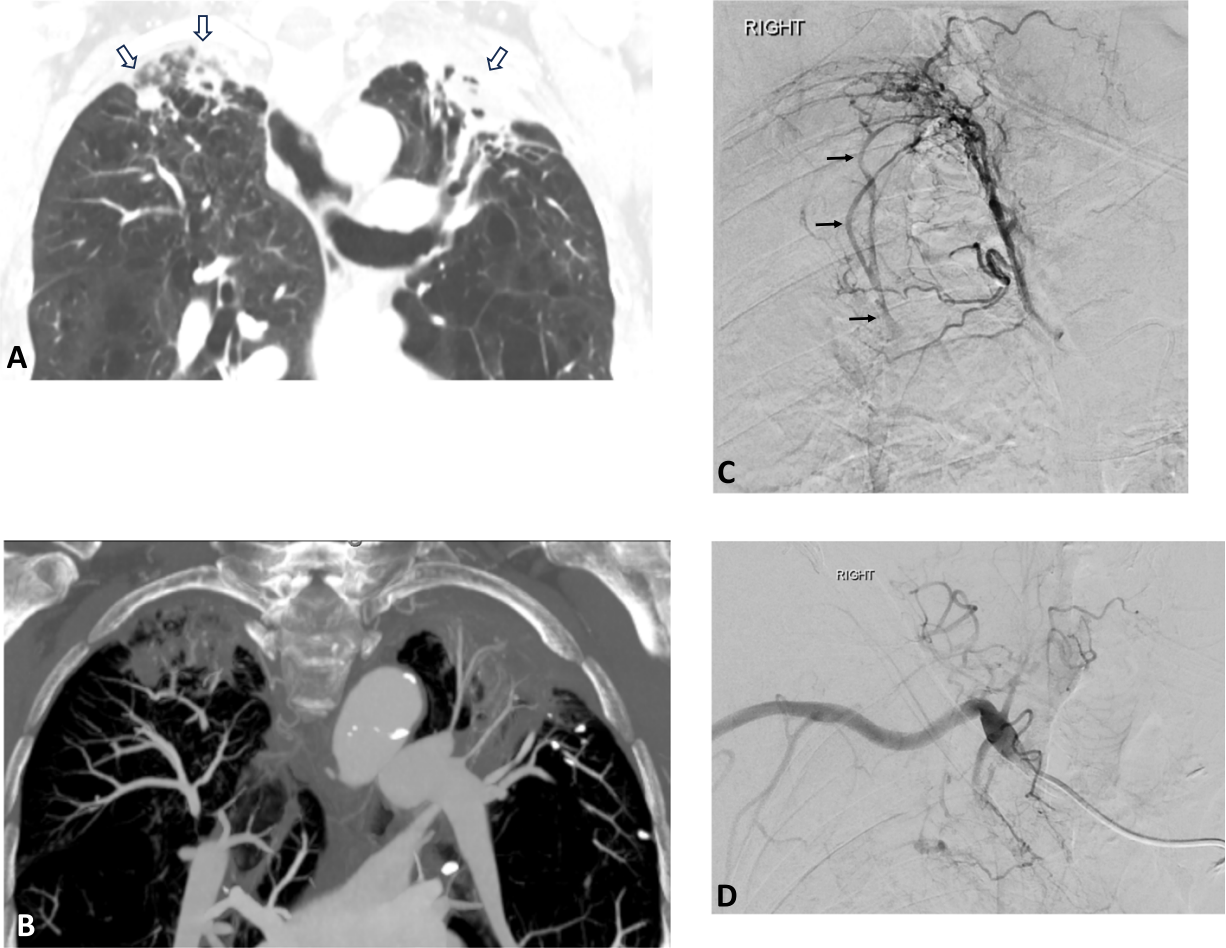
72-year-old woman with post-primary TB presenting with worsening haemoptysis. **A** Coronal CT image of the upper zones shows bilateral fibrotic changes and apical cavitations (open arrows). **B** Maximum intensity projection coronal CT image shows abnormal vessel recruitment by the apical inflammatory mycetomas. **C** Selective angiogram of the R intercostobronchial trunk shows recruitment of multiple intercostal arteries and florid neo-angiogenesis. The black arrows represent intercostal artery-to-pulmonary venous shunting. **D** Selective right subclavian angiogram demonstrates further vessel recruitment to the apical cavity

Aspergillomas are a type of mycetoma or fungal ball, and typically occur in patients with pre-existing lung cavitation. They are most common in post-primary tuberculosis (TB) and sarcoidosis, as was in this series. The cavity wall consists of acute on chronic inflammatory infiltrate and granulation tissue and cause florid neo-angiogenesis and recruitment of large number of collaterals including the bronchial and non-bronchial arterial circulation (Fig. 3). The disease process may also invade directly through pulmonary/bronchial vessels, resulting in haemoptysis [45, 46]. The findings further highlight the poor outcomes for patients with aspergilloma and the need for concurrent and aggressive anti-fungal treatment.

### Neurological complications

The number of ischaemic strokes (6.4%) in this cohort was higher than has been previously cited in the literature (up to 2%) [4].

#### Pre-embolisation

In 2 of the 9 patients who suffered peri-procedural stroke, neurological deficit manifested prior to embolisation, and the procedures were abandoned. Both patients had imaging confirmation of anterior circulation ischaemic infarction. In one patient, it was technically not possible to cannulate the target bronchial artery, and the second patient became haemodynamically unstable before target arteries were selected. Given that embolisation had yet to be performed it is speculated the underlying mechanism was prolonged manipulation in the aortic arch which may have resulted in embolisation of thrombus or plaque into the cerebral circulation.

#### Post embolisation

Four of the 9 patients who suffered a stroke presented with cavitating upper lobe infections either due to aspergilloma or non-tuberculous pneumonia. In these patients, the subclavian artery and its cervical branches were interrogated for involvement. The potential mechanism for stroke in these cases is speculated to be due to abnormal arterial branches resulting from the underlying inflammatory process.

Non-target embolisation may occur through abnormal communications between the bronchial or subclavian ateries with the vertebral artery, or via abnormal shunting between systemic arteries and a pulmonary vein. Indeed, shunting between the bronchial/non-bronchial systemic arteries and a pulmonary vein was identified on angiography in 2 of these 4 patients. When these communications were identified, the preferred embolic agent of ns-PVA was substituted for the more viscous preparations of NBCA or EVOH, in attempts to limit the degree of distal embolisation. EVOH or NBCA would be injected under direct fluoroscopic guidance and injection ceased prior to any embolic entering the pulmonary vein.

Despite adapting the choice of embolic agent, both of these patients developed embolic stroke. In one patient, cervical branches of the right subclavian artery were embolised with Onyx with subsequent diplopia and MRI confirmation of cerebellar infarct. In another, intercostal branches were embolised with NBCA (1:3 dilution with lipiodol) and unsuccessful attempts were made to superselect cervical branches of the right subclavian artery supplying an apical cavity. The patient developed a dense left hemiplegia and CTA confirmed right M2 segment thrombosis unsuitable for thrombectomy.

Notably, there were no cases of anterior spinal ischaemia in the cohort. This is the most feared complication of BAE and operators take utmost care to look for and avoid non-target embolisation. The authors postulate that embolic stroke is under-reported in comparison to anterior spinal ischaemia.

### Pre-BAE CT

Whilst the data did not demonstrate a significant impact of MDCTA on improving technical success of BAE, there are clear benefits to performing MDCTA. As well as identifying target arteries for embolisation, availability of pre-procedural MDCTA permits assessment of the underlying cause, procedure planning and additional cardiorespiratory factors that may preclude BAE [7]. It may be argued that the aortic arch and proximal thoracic aorta should be assessed for the degree of atherosclerotic disease to guide discussions regarding stroke risk during informed consent. However, if MDCTA is not readily available, it should not delay prompt therapeutic embolisation [4, 16].

### Limitations

Firstly, retrospective data collection is associated with selection bias. Secondly, 16 patients were lost to follow-up and therefore excluded from analysis. Many of these patients were repatriated to their local centres and knowledge of their outcomes may have impacted the clinical success and recurrence rates.

## Conclusions

BAE is an effective endovascular treatment in patients with massive and recurrent haemoptysis. However, there is a well-documented risk of recurrent symptoms and early mortality, particularly in the setting of aspergilloma and non-tuberculous pneumonia.

This study did not demonstrate improvement in technical success with pre-procedural MDCTA. Nonetheless it is recommended to allow identification of the underlying aetiology, assessment of the bronchial arteries, presence of non-systemic collaterals and assessment of underlying arterial disease precluding BAE which may increase the risk of embolic stroke.

Finally, the risk of stroke should not be underestimated. Stroke may result from prolonged catheter manipulation in the aortic arch or due to non-target embolisation from systemic-pulmonary shunts which may not be visible on angiography. Patients should be counselled appropriately during informed consent prior to embarking on BAE.

## Data Availability

The datasets used and/or analysed during the current study are available from the corresponding author on reasonable request.
